# Acute Rheumatic Fever in Children: The Known and the Unknown Among Saudi Mothers in the Jazan Region

**DOI:** 10.7759/cureus.56349

**Published:** 2024-03-17

**Authors:** Ebtihal E Eltyeb, Sami A Alhazmi, Abdulrahman M Darraj, Adeeb H Alibrahim, Mohammad A Alhazmi, Mohammed A Muafa, Fanan A Hakami, Ikhlas I Daak, Rania Q Majrabi

**Affiliations:** 1 Faculty of Medicine, Jazan University, Jazan, SAU

**Keywords:** saudi, knowledge, children, attitude, acute rheumatic fever

## Abstract

Background

Acute rheumatic fever (ARF) is a significant public health problem that causes morbidity and mortality in low and middle-income countries. Therefore, this study aims to assess mothers' knowledge of acute rheumatic fever and their attitudes in the Jazan region.

Methods

A cross-sectional study was conducted between the mothers using an online survey. The knowledge level was ranked as poor, fair, and good. In contrast, the attitudes were ranked as positive or negative. Association with sociodemographic variables was assessed, and statistical significance was set at *p* < 0.05.

Results

Four hundred thirty-six (436) mothers were included; 39.9% of them were aged 21-30 years, 68.8% were married, 57.3% were non-workers, and 72.2% were university-educated. Most of the mothers had a poor level of knowledge (53%); however, positive attitudes toward the disease were reported in 79.1% of mothers. The poor knowledge levels were related to age, marital status, occupation, and monthly income.

Conclusion

Despite having positive attitudes toward diagnosing and managing ARF, most of the mothers showed poor knowledge of acute rheumatic fever. This study highlights the situation in the Jazan region, which could be an essential basis for constructing an educational program to raise awareness and knowledge of acute rheumatic fever in the community.

## Introduction

Acute rheumatic fever (ARF) is a disease that results from an autoimmune reaction to a throat or skin infection (impetigo) with the group A Streptococcus (GAS) bacteria affecting children in the five to 14-year range [1]. The Jones criteria outline the clinical presentations of ARF for the diagnosis [2]. These symptoms and signs are arthritis involving the large joints, cardiac valvular disorders, involuntary movements (Sydenham's chorea), and skin and subcutaneous manifestations. Rheumatic heart disease (RHD), the most common cause of heart failure in poor populations, is a long-term complication of ARF [3]. The primary prevention of ARF has focused on appropriate antibiotic treatment of symptomatic bacterial pharyngitis prescribed by doctors; however, about one-third of ARF results from non-apparent streptococcal infections [4,5].

Although the incidence of ARF has dramatically decreased in developed nations, it has not yet been completely eradicated. Still, it poses a significant health burden in developing countries and has terrible long-term consequences. The annual incidence rate is comparatively larger in Asia, Australasia, and the Middle East (>10/100,000) than it is in America and Western European countries (<10/100,000) [6]. In Saudi Arabia, data concerning the prevalence of ARF and RHD are few; nevertheless, the percentage of children with RHD in Saudi Arabia, as reported by one study, continued to be high, reaching 3.1 per 1000 school-aged children despite the advancement in the socioeconomic evolution of the country [7,8].

Maternal perception and attitudes toward ARF help diagnose and manage the affected children. The first defense against ARF is primary prevention, which involves diagnosing and treating pharyngitis with the appropriate medications. Early access to primary healthcare facilities, compliance with therapy, and avoidance of irrational antibiotic use are crucial for reducing the incidence of this disease [9]. At the same time, secondary prevention is being practiced in patients who experience ARF symptoms to avoid carditis by using equivalent antibiotic options in pharyngitis [10,11].

This study assessed mothers' knowledge and attitudes regarding acute rheumatic fever in the Jazan region.

## Materials and methods

A cross-sectional study was performed in the Jazan region southwest of the Kingdom of Saudi Arabia (KSA). This port city is one of the 13 regions in the Kingdom. Jazan is considered the second-smallest province in KSA, with a population of 1,637,361, according to the 2019 census [12]. The study was carried out from June 2023 to August 2023.

The study population included all mothers living in the Jazan region who agreed to participate in and complete the survey and excluded mothers living outside the Jazan region or those who did not agree to or complete the study. According to the General Authority for Statistics, the total population of Jazan is 1,637,361, and the number of females in the Jazan region is 748,857, according to the latest data issued in 2019 [12]. The sample size was estimated using the Raosoft calculator (Raosoft Inc., Seattle, WA, US), which utilized a 95% confidence interval, 5% error margin, and 50% anticipated response. The calculated sample size was 384, which increased to 422, assuming the non-response rate would be 10%.

Data were collected using an online self-administrated survey designed in Google Forms (Google LLC, Mountain View, California, United States). The simple random sampling technique for the coastal, mountain, and plain regions of Jazan, reflecting all regions of Jazan, was used as the collection method in this study. Before distributing the questionnaire, a pilot study including 20 participants was performed to assess the questions' length and clarity, any language mistakes, and the time needed to complete the questionnaire. In the reliability check, Cronbach's α value was 0.931, and we considered the questionnaire internally consistent.

The questionnaire was prepared and designed in Arabic to suit the participants. The survey was modified from previous studies [13,14] and was composed of three parts; the first part assessed the demographic variables of the participants (age, residence, occupation, monthly income, education, marital status, and the number of children), the second part contained 14 questions with the response - yes, no, or I do not know to assess the participants' levels of comprehension of rheumatic fever regarding general information, symptoms, and signs, complications, and treatment. The last part of the questionnaire measured the participants' attitude toward rheumatic fever and contained five items using a three-point Likert scale.

Data were analyzed using the SPSS statistical program version 23 (IBM Corp, Armonk, NY, USA). Descriptive and inferential statistics involve the Pearson chi-squared test to evaluate the association between the knowledge score and different variables. 

One-way analysis of variance (ANOVA) and independent t-tests were employed to confirm whether there were any statistically significant disparities between the mean knowledge scores of two or more independent groups. Statistical significance was considered with a p-value of < 0.05.

Score

The questionnaire consisted of 14 questions assessing mothers' ARF knowledge. The questions had the options of Yes, No, and Do not know. There were seven questions with the correct answer Yes, and the remaining ones with the correct answer No. For each question, one point was given for each correct answer, -1 for the wrong answer, and zero for Do not know, resulting in a score range of -14 to 14, which was then converted into a percentage. A mother's knowledge level is considered fair if they scored between 50% and 75%, indicating that they have identified more than half of the correct answers. On the other hand, above this score is considered good, and below it is considered poor.

The level of the mothers' attitude was measured using the three-point Likert scale, with one indicating less importance and three indicating more significant importance. The attitude score ranged between -1 and 1; the average score items were then converted to a percentage using the formula (Mean-1)*25 and rated as positive above 50% and negative below 50%.

## Results

In this study, 436 mothers were included; 39.9% were aged 21-30, 68.8% were married, 65.8% were from rural areas in Jazan, and 72.2% had a university level of education. Of the participants, 57.3% were non-workers compared to 32.6% who were public sector workers, with 51.8% having an income of less than 5000 Saudi Riyals, as shown in Table 1.

**Table 1:** Characteristics of participant mothers (n = 436) SR = Saudi Riyal, % = percentage, n = number

	Number	Percent (%)
Age (years)		
< 20	61	14
21 - 30	174	39.9
31 -40	127	29.1
>40	74	17
Area of residence		
Jazan (urban )	149	34.2
Jazan (rural)	287	65.8
Educational level		
Primary school	8	1.8
Intermediate school	23	5.3
Secondary school	85	19.5
University	315	72.2
Non-educated	5	1.1
Occupation		
Not work	250	57.3
Work in the public sector	142	32.6
Work in the private sector	44	10.1
Income (monthly/SR)		
<5000	226	51.8
5000-10000	117	26.8
10001-20000	79	18.1
>20000	14	3.2
Marital status		
Married	300	68.8
Single	107	24.5
Divorced	21	4.8
Widow	8	1.8
Number of children		
No children	137	31.4
1-3	190	43.6
4-7	89	20.4
>7	20	4.6

Table 2 shows that 37.2% of the mothers knew that ARF is related to throat infection, 40.1% knew the symptoms of ARF with joint pain, heart diseases, skin rash, and involuntary movement while 32.8% of the mothers related ARF to heart valve disease as a complication; however, more than half of the mothers' answers (do not know) were in all ranges of the items.

**Table 2:** Mothers' knowledge regarding acute rheumatic fever (n = 436) *Indicates that Yes is the correct answer, ** indicates that No is the correct answer ARF = acute rheumatic fever, n = number

Knowledge Variable	Yes	No	Don’t know
ARF is an infectious disease**	44 (10.1%)	129 (29.6%)	263 (60.3%)
ARF is an inherited disease**	65 (14.9%)	91 (20.9%)	280 (64.2%)
ARF cannot be diagnosed in children**	32 (7.3%)	145 (33.3%)	259 (59.4%)
ARF is associated with throat infection*	162 (37.2%)	28 (6.4%)	246 (56.4%)
Both viral and bacterial throat infection is associated with ARF**	89 (20.4%)	82 (18.8%)	256 (60.8%)
Joint pain, heart diseases, skin rash, and involuntary movement are symptoms related to ARF*	175 (40.1%)	11 (2.5%)	250 (57.3%)
The presence of fever is necessary to diagnose ARF**	100 (22.9%)	51 (11.7%)	285 (65.4%)
Destruction of heart valves is one complication of ARF*	143 (32.8%)	9 (2.1%)	284 (65.1%)
Permanent joint destruction is a complication of ARF*	157 (36%)	18 (4.1%)	261 (59.9%)
ARF is a fatal disease*	127 (29.1%)	28 (6.4%)	281 (64.4%)
The previous occurrence of ARF gives the affected person long-life immunity to the disease to prevent its recurrence**	57 (13.1%)	56 (12.8%)	323 (74.1%)
ARF needs doctor consultation and management*	193 (44.3%)	14 (3.2%)	229 (52.5%)
ARF can be treated at home with herbal medicine**	25 (5.7%)	144 (33%)	267 (61.2%)
The optimal period of antibiotic therapy for throat infection can protect from ARF*	145 (33.3%)	30 (6.9%)	261 (59.9%)

**Figure 1:**
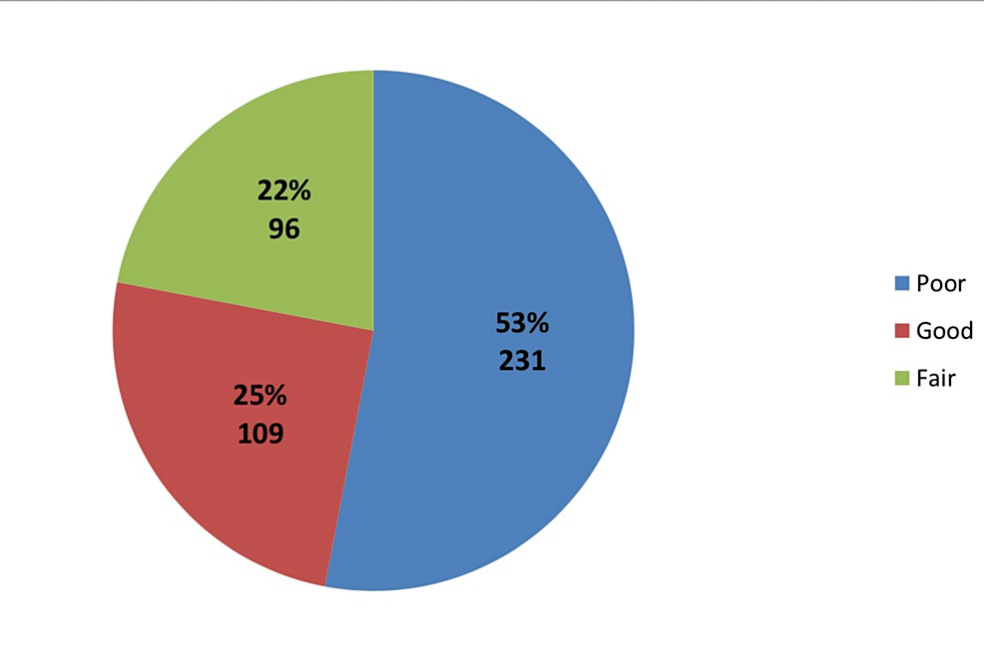
Mothers' knowledge regarding acute rheumatic fever (n = 436) % = percentage The knowledge level is considered good if the score is >75%, fair 50-75%, and poor <50%.

As shown in Figure 1, more than half of the mothers have poor knowledge regarding ARF, compared to only 25% who have good knowledge. The minimum knowledge score was -2 while the maximum was 14, with a knowledge mean of 2.9± 3.6. About 42.7% of mothers have a score of zero, and only 2% have a score of 14.

A chi-square test was performed to compare the levels of knowledge among the mothers' sociodemographic variables. As shown in Table 3, the knowledge level reported an age difference (p-value 0.024), with the age group 21-30 reporting more poor knowledge than other groups. Additionally, occupations with non-workers and monthly incomes of less than 5000 SR were found to show a significant difference (p-value 0.011) from different categories in terms of a poor level of knowledge. Also, marital status reported a significant difference (p-value 0.035) between the mothers with married status, which influenced the level of knowledge. However, one-way ANOVA showed no statistically significant differences between the mean knowledge scores of the independent groups.

**Table 3:** Level of knowledge difference related to the mothers’ sociodemographic variables *p-value is significant at <0.05. SR = Saudi Riyal, % = percentage, n = number Knowledge level is considered good if the score is >75%, fair 50-75%, and poor <50%.

Factor	Level of knowledge			p-value
	Poor( n = 230)	Fair (n = 98)	good (n = 107 )	
Age group				
< 20	44	9	8	0.024*
21 - 30	86	44	44	
31 - 40	59	27	40	
>40	41	18	15	
Area of residence				
Jazan (urban)	76	32	40	0.883
Jazan (rural)	154	66	67	
Educational level					
Primary school	4	3	1	0.087
Intermediate school	17	4	2	
Secondary school	55	13	17	
University	151	77	86	
Non-educated	3	1	1	
Occupation				
Not work	136	64	50	0.011*
Work in the public sector	65	29	47	
Work in the private sector	29	5	10	
Income (monthly/SR)				
<5000	134	44	48	0.011*
5000-10000	51	35	30	
10001-20000	37	14	28	
>20000	8	5	1	
Marital status				
Married	142	73	84	0.035*
Single	69	18	20	
Divorced	13	6	2	
Widow	6	1	1	
Number of children				
No children	86	24	27	0.100
1-3	88	49	53	
4-7	43	21	24	
>7	13	4	3	

The attitude statements included five items, which are the importance of physician consultation in any throat infection, completion of the recommended treatment period, using traditional home remedy and herbal medication for treating pharyngitis, urgent doctor consultation if any ARF-related symptom was present, and the importance of increasing knowledge about the seriousness of throat infection and related complications. The mothers' attitudes regarding ARF were positive, with a mean of 1.79±0.4. The positive attitude constituted 79.1% among the mothers and was significantly related to the monthly income (p-value = 0.008), as shown in Table 4.

**Table 4:** Level of attitude difference related to the mothers’ sociodemographic variables *p-value is significant at <0.05 SR = Saudi Riyal, n = number Attitude is considered positive above 50% and negative below 50%.

Variable	Level of Attitude		p-value
	Negative( n = 91)	Positive(n = 345)	
Age group			
<20	13	48	0.314
21 - 30	29	145	
31 - 40	30	96	
>40	19	55	
Area of residence			
Jazan (urban)	37	111	0.316
Jazan (rural)	54	233	
Educational level				
Primary school	2	6	0.813
Intermediate school	7	16	
Secondary school	16	69	
University	65	249	
Non-educated	1	4	
Occupation			
Not work	48	202	0.445
Work in the public sector	31	110	
Work in the private sector	12	32	
Income (monthly/SR)			
<5000	39	187	0.008*
5000-10000	31	85	
10001-20000	14	65	
>20000	7	7	
Marital status			
Married	67	232	0.640
Single	19	88	
Divorced	3	18	
Widow	2	6	
Number of children			
No children	24	113	0.327
1-3	45	145	
4-7	20	68	
>7	2	18	

## Discussion

There are many strategies to lessen the effects of ARF, such as primary prevention, which involves diagnosing pharyngitis and treating it with the proper medications [15]. Lack of knowledge about ARF remains the main barrier to improving disease prevention and management results. Therefore, this study assessed mothers' knowledge and attitudes regarding ARF in the Jazan region of Saudi Arabia.

The mothers in this study showed poor knowledge regarding the diagnosis and treatment of ARF. Only 25% of participants had good knowledge, compared to more than half who had poor knowledge. These results are more consistent with the recent Saudi study conducted in Riyadh, indicating that there is generally poor knowledge in the Saudi community. More than two-thirds of the study participants do not relate ARF to sore throat and are unaware of the potential risk of untreated pharyngitis in children [13]. Another study was conducted in the Aseer region of Saudi Arabia and reported a significant lack of knowledge that reached 52.9%, specifically among younger participants [16]. Another study with 1,596 participants from all regions of Saudi Arabia showed that age, gender, and occupation substantially impacted participants' attitudes and level of knowledge [14]. In contrast to our study, the degree of knowledge exhibits a higher score; nonetheless, the two studies' levels of attitudes and influencing factors were similar.

About a third of the mothers in the current study knew that ARF is not infectious, and more than one-third (37.2%) knew it was strongly related to pharyngitis. However, 23% of participants thought fever was necessary to diagnose ARF. Although fever is one of the minor criteria that help diagnose ARF, the absence of fever cannot exclude the diagnosis of the disease according to Jones Criteria [3]. Additionally, 40% of the mothers were aware of the symptoms of the disease, and about a third of them knew the complications that involved heart valves and joints. However, only 20.3% knew that taking antibiotics for a sore throat prevents ARF. According to available data, these mothers' concepts were consistent with previous national studies [13,14,16]. The poor knowledge levels among the mothers in this study showed a significant relation to the age group 21-30, married, non-worker mothers, and low monthly income. This result matched an African study in Cameroon, where inadequate knowledge of ARH is strongly associated with age and occupation [17].

The mothers' attitude toward ARF diagnosis and management of pharyngitis showed a positive attitude, with more than 79% of mothers indicating the importance of physician consultation and completion of the recommended treatment period. Although close to one-fifth of the mothers agreed that the traditional home remedy and herbal medication are good options for treating pharyngitis, about 90% of the participants indicated needing urgent doctor consultation if any ARF-related symptom was present. This proportion was very close to that reported in the Saudi studies, demonstrating a positive attitude among the participants as compared to the poor knowledge [13,14].

Although this study provided fundamental insights into mothers' attitudes and knowledge of ARH in the Jazan area, several limitations must be considered. The self-reported online survey is known to be biased in reporting and recall. Additionally, the 'do not know' responses are calculated as zero, which makes it difficult to differentiate between mothers' poor knowledge and their preference not to answer. Lastly, we need to include additional community sectors to improve the study's generalizability.

## Conclusions

Acute rheumatic fever is a severe health issue that causes morbidity and mortality worldwide. Despite significant medical and social advancements, the disease is still not completely eradicated. The mothers’ comprehension of the disease in this study showed suboptimal knowledge that was influenced by age, marital status, occupation, and monthly income; however, positive attitudes toward the disease were reported between them. Therefore, one key strategy to help eradicate ARF globally will be raising community awareness through ongoing integrated health education programs to prevent and cure streptococcal infections as soon as they arise. It is believed that highlighting the continuing global issue of ARF will encourage more research into strategies to lessen the impact of this illness.
